# Using digital assessment technology to detect neuropsychological problems in primary care settings

**DOI:** 10.3389/fpsyg.2023.1280593

**Published:** 2023-11-17

**Authors:** David J. Libon, Emily Frances Matusz, Stephanie Cosentino, Catherine C. Price, Rod Swenson, Meagan Vermeulen, Terrie Beth Ginsberg, Adaora Obiageli Okoli-Umeweni, Leonard Powell, Robert Nagele, Sean Tobyne, Joyce Rios Gomes-Osman, Alvaro Pascual-Leone

**Affiliations:** ^1^School of Osteopathic Medicine, New Jersey Institute for Successful Aging, Rowan University, Stratford, NJ, United States; ^2^Department of Psychology, Rowan University, Glassboro, NJ, United States; ^3^Department of Clinical and Health Psychology, University of Florida, Gainesville, FL, United States; ^4^Cognitive Neuroscience Division, Department of Neurology, Taub Institute and Sergievsky Center, Columbia University Medical Center, New York, NY, United States; ^5^School of Medicine and Health Sciences, University of North Dakota, Grand Forks, ND, United States; ^6^Department of Family Practice, School of Osteopathic Medicine, Rowan University, Stratford, NJ, United States; ^7^Linus Health, Boston, MA, United States; ^8^Sidney Wolk Center for Memory Health, and Eleanor and Herbert Bearak Memory Wellness for Life Program, Hinda and Arthur Marcus Institute for Aging Research and Deanna, Hebrew SeniorLife, Boston, MA, United States; ^9^Department of Neurology and Harvard Medical School, Boston, MA, United States

**Keywords:** clock drawing, executive control, episodic memory, mild cognitive impairment, Alzheimer’s disease, Mini-Cog

## Abstract

**Introduction:**

Screening for neurocognitive impairment and psychological distress in ambulatory primary and specialty care medical settings is an increasing necessity. The Core Cognitive Evaluation™ (CCE) is administered/scored using an iPad, requires approximately 8 min, assesses 3- word free recall and clock drawing to command and copy, asks questions about lifestyle and health, and queries for psychological distress. This information is linked with patients’ self- reported concerns about memory and their cardiovascular risks.

**Methods:**

A total of 199 ambulatory patients were screened with the CCE as part of their routine medical care. The CCE provides several summary indices, and scores on 44 individual digital clock variables across command and copy tests conditions.

**Results:**

Subjective memory concerns were endorsed by 41% of participants. Approximately 31% of participants reported psychological distress involving loneliness, anxiety, or depression. Patients with self-reported memory concerns scored lower on a combined delay 3- word/ clock drawing index (*p* < 0.016), the total summary clock drawing command/ copy score (*p* < 0.050), and clock drawing to command Drawing Efficiency (*p* < 0.036) and Simple and Complex Motor (*p* < 0.029) indices. Patients treated for diabetes and atherosclerotic cardiovascular disease (ASCVD) scored lower on selected CCE outcome measures (*p* < 0.035). Factor analyses suggest that approximately 10 underlying variables can explain digital clock drawing performance.

**Discussion:**

The CCE is a powerful neurocognitive assessment tool that is sensitive to patient’s subjective concerns about possible decline in memory, mood symptoms, possible cognitive impairment, and cardiovascular risk. iPad administration ensures total reliability for test administration and scoring. The CCE is easily deployable in outpatient ambulatory primary care settings.

## Introduction

Current estimates suggest that approximately 16% of the US population over the age of 65 suffers with mild cognitive impairment (MCI; American Academy of Neurology [AAN], 2017). It has been suggested that within 2 years many who are diagnosed with MCI will go on to develop dementia (Petersen et al., 2018). Moreover, approximately 6.2 million Americans, aged 65 and older have Alzheimer’s disease (AD; El-Hayek et al., 2019; Alzheimer’s Association, 2023). Past research suggests that the neuropathology underlying AD likely begins decades before the emergence of obvious clinical symptoms (Hulette et al., 1998; Price and Morris, 1999; Gallardo and Holtzman, 2019). Newer pharmacological therapies to treat ADRD have been deployed and continue to be developed (van Dyck et al., 2023). Although these therapies are promising, it is universally acknowledged that early diagnosis and characterization of ADRD is key to optimize the effectiveness of pharmacological agents and lifestyle modifications aimed at improving brain health. The current diagnostic paradigm used to characterize and potentially diagnose ADRD involves specialized neuropsychological assessment, brain imaging studies, and an analyses of serum biomarkers. However, many of these procedures are expensive, invasive, not readily available, and rely on highly specialized healthcare professionals.

In addition to cognitive decline, initial symptoms of emergent ADRD can also revolve around the presence of symptoms of psychological distress such as depression and anxiety (Mendez, 2021; Jiang et al., 2022), as well as patients’ self-reported or subjective complaint of memory and memory-related cognitive decline (Jonker et al., 2000; Chapman et al., 2021; Morrison and Oliver, 2023). Indeed, increasing evidence suggests that subjective cognitive decline (SCD) is a risk for AD (Chapman et al., 2021). Moreover, common cardiovascular problems such as hypertension, hyperlipidemia, diabetes, current tobacco use, and atherosclerotic cardiovascular disease are well- known to potentiate the emergence of ADRD (Emrani et al., 2020; Vintimilla et al., 2022).

Yet, an assessment of psychological distress and self-perceived memory problems in the context of cardiovascular risk factors is absent from traditional paper and pencil tests used for cognitive screening such as the mini-mental state examination (Folstein et al., 1975) and the Montreal Cognitive Assessment (Nasreddine et al., 2005). Also, most individuals ultimately diagnosed and treated for ADRD are often seen in specialty clinics, usually urban university-affiliated outpatient memory clinics. As such, people living in rural areas have less access to much needed assessment and diagnostic services. Thus, to optimize ADRD screening, there is a need for a test that is reliable and easy to use; able to measure and flag cognitive impairment and psychological distress in the context of medical risk factors associated with ADRD; and can be deployed in both primary ambulatory and specialty outpatient healthcare settings.

The Core Cognitive Evaluation™ (CCE) is administered and scored using an iPad. It is modeled after the paper and pencil version of the Mini-Cog (Borson et al., 2003). The CCE takes approximately 8 min to digitally administer and score. The CCE assesses 3-word immediate and delayed free recall; clock drawing to command and copy (Libon et al., 1993, 1996; Cosentino et al., 2004); and a set of questions about lifestyle, health, and psychological distress. The CCE leverages a digital version of the clock drawing test (CDT) and is developed to enhance sensitivity to subtle cognitive deficits that may not be picked up using traditional paper and pencil assessment procedures (Libon et al., 2022). Indeed, the DCTclock™, a digitized version of the clock drawing test that also leverages artificial intelligence, can detect, automatically score, and operationally define behavior previously unobtainable using traditional paper and pencil screening measures. It is also associated with critical neuropsychological parameters associated with ADRD. For example, Matusz et al. (2023) demonstrated that DCTclock performance was able to distinguish between groups defined as cognitively normal (CN) versus subtle cognitive impairment (SCI; Edmonds et al., 2015), amnestic mild cognitive impairment (Bondi et al., 2014), and mixed/dysexecutive mild cognitive impairment (Bondi et al., 2014). Dion et al. (2020) showed that longer intra-component latencies obtained with the digital clock drawing test (dCDT, e.g., elapsed time between the production of the clock face and the next pen stroke) were associated with worse performance on neuropsychological tests measuring working memory and Information Processing Speed. In another study, Dion et al. (2022) found that number misplacement on DCTclock was negatively associated with semantic, visuospatial, and visuoconstructional operations as well as connectivity from the basal nucleus of Meynert to the anterior cingulate cortex. Finally, Rentz et al. (2021) studied a group of Harvard Aging Brain Study participants. Among participants assessed as cognitively normal who were also positive for ADRD- related pathology based on PET imaging, DCTclock performance was associated with greater amyloid and tau burden and showed better group discrimination than other neuropsychological tests. Thus, past research demonstrates that DCTclock is sensitive to behavior not otherwise observable and neuropsychological and neuropathological markers that are well-known to be associated with ADRD.

In the current research, the CCE was administered to ambulatory, primary care patients. The purpose of the current research was two-fold. First, this study aimed to assess how well the CCE can measure cognitive abilities and symptoms to suggest psychological distress; how these features may be linked to patients’ subjective complaint of memory and memory-related cognitive decline; and how the CCE may be associated with common cardiovascular problems such as hypertension, hyperlipidemia, diabetes, current tobacco use, and atherosclerotic cardiovascular disease. This study examined the hypothesis that poorer performance on the CCE would be associated with greater memory concerns, psychological distress, and cardiovascular risk factors. The second goal of the study was to extract the core features of command and copy clock drawing performance using a series of factor analyses.

## Materials and methods

### Participants

All participants (*n* = 199) taking part in this research were receiving routine medical care and were recruited from the Rowan University, Department of Family Medicine (*n* = 57) and the New Jersey Institute for Successful Aging (*n* = 142; 65.40% female). Exclusion criteria included a clinical diagnosis of mild cognitive impairment (MCI) or dementia, and if English was not the participants first language. All patients were well known to their treating physician and were assessed yearly. The absence of MCI or dementia was determined with an in-depth clinical interview and the Mini Mental State Examination or the Montrel Cognitive Assessment. The Rowan University Institutional Review Board approved this study; all participants provided written consent consistent with the Declaration of Helsinki.

### iPad digital assessment

All testing was obtained using a 12-inch iPad and an accompanying Apple Pencil. A trained examiner proctored the test; however, all test instructions were delivered verbally by the iPad. During the assessment procedure, the iPad was kept in the portrait position. The assessment began by telling the participant that they will hear three words and that after the words were delivered, the participant is asked to repeat and remember the three words. Three-word immediate free recall was followed by clock drawing to command where the participant was asked “to draw the face of a clock, put in all of the numbers, and set the hands for ‘*10 after 11*.”’ This was followed by the clock drawing copy test condition where the participant sees a model of a clock with hands set for “*10 after 11*” and is asked to copy the model in the provided space. After the completion of the clock drawing copy test condition, the participant is asked to recall the 3 words previously presented. Mean CCE assessment time was 8.05 s (sd = 1.62; range 5.05–12.07 s).

As described by Matusz et al. (2023) the DCTclock is based on the traditional paper and pencil clock drawing task and was originally designed with cooperation from the Clock Sketch Consortium (Libon et al., 2014) and colleagues from the Lahey Clinic and the Massachusetts Institute of Technology. The DCTclock was developed and licensed for research by Digital Cognition Technologies Inc., now part of Linus Health, Inc. The DCTclock is a class II medical device listed by the Food and Drug Administration (FDA) for cognitive assessment. As described above, all clock drawings were obtained using an iPad and Apple Pencil.

The DCTclock produces numerous objective measurements derived from approximately 5,000 digital clock drawings using machine learning algorithms (Davis et al., 2014; Binaco et al., 2020; Matusz et al., 2023). Machine learning algorithms were previously developed to calculate meaningful clock scores based on their ability to discriminate performance between thousands of healthy controls and participants from different diagnostic groups, including amnestic MCI, AD, Parkinson’s disease (PD), and other neurodegenerative disorders (Davis et al., 2014; Souillard-Mandar et al., 2016, 2021).

The DCTclock algorithms have been previously described by Souillard-Mandar et al. (2016, 2021). Briefly, data analyzed through the DCTclock algorithms produces a total combined DCTclock command/ copy summary score (range 0–100), and four command and four copy index scores that assess Drawing Efficiency, Simple and Complex Motor Operations, Information Processing Speed, and Spatial Reasoning. Index scores are expressed as z- scores with a mean of 0 and a standard deviation of 1.0. Table 1 contains a description of all 9 DCTclock indices. Listed in Supplementary Table 1 are the specific variables that underlie each of these four command/ copy clock indices. The variables underlying the command or copy clock are separately combined in a weighted linear model to produce the four index scores for each clock test condition. The eight total index scores (four from command, four from copy) are combined in a weighted linear model to produce the DCTclock total combined command/ copy score, ranging from 0–100 and a predicted risk of cognitive impairment.

**TABLE 1:** Digital clock drawing outcome variables.

Total score	A single score between 0 and 100 that captures overall performance across command and copy conditions
**Index scales (command and copy)**	
Drawing Efficiency	The efficiency the participant demonstrated during the process of drawing the clock. This considers metrics such as total time spent compared to amount of ink used, pen strokes and ink length, size of the drawing, etc.
Simple/Complex Motor Operations	The motor components involved during the process of drawing the clock. This considers metrics including speed and oscillatory motion and can be helpful in parsing out graphomotor concerns.
Information Processing Speed	The ability to process information demonstrated during the process of drawing the clock. This considers metrics including latencies, pauses, and relative time spent thinking (without pen to paper) versus actively drawing.
Spatial Reasoning	The spatial abilities demonstrated during the process of drawing the clock. This considers metrics including geometric and spatial placement of the various properties of the drawing.

Score calculation is automated, and cloud based. Composite and subscale scores are calculated for both command and copy conditions and normed with respect to cognitively healthy individuals. Composite scales and subscale metrics are adjusted for age.

### CCE outcome variables

#### Three-word recall

Outcome variables included the total number of immediate and delay- free words that were recalled.

#### DCTclock command/copy score (range 0–100) and the Digital Clock And Recall score (range 0–5)

As noted above, the total command and copy clock drawing performance is scored on a scale of 0–100. First, DCTclock performance is assigned an integer value based upon the DCT score falling within three different bands: 0–60 is assigned 0 points, 61–74 is assigned 1 point, and 75–100 is assigned 2 points. The clock summary score is summed with the number of words correctly recalled after a delay, with a maximum score of 3, to produce the Digital Clock and Recall (DCR™) Score ranging from 0 to 5.

Scores ranging 0–1 were considered indicative of cognitive impairment, scores between 2 and 3 were considered borderline for cognitive impairment, and scores 4–5 were considered negative screening of cognitive impairment. Automatic speech recognition (ASR) is used to transcribe recordings of patient vocalizations, which are then compared against the 3 expected words dictated to the patient during the immediate repeat portion of the DCR. Upon the conclusion of the CCE, all results are immediately and automatically scored by software, and a report is immediately available to the clinician.

#### Psychological distress

Upon concluding the delayed 3- word test condition, participants were assessed with a series of 32 yes/ no questions about their health and lifestyle. The participant was asked to read all questions to themselves, and using the Apple Pencil to tic “yes” or “no.” The three CCE yes/no questions analyzed in the current research that assessed for psychological distress included: “Sometimes I feel as though I am alone and lack support” (loneliness), “I have felt anxious, nervous, or worried in the last month” (anxiety), and “During the past month I have often been bothered by feeling down, depressed, hopeless, little interest or pleasure in doing things” (depression). Questions regarding loneliness, anxiety, and depression were summed to create a total psychological distress score (range 0–3).

#### Self-reported memory concern

Patients were asked “Do you have concerns about your memory or thinking abilities.” This question was scored 1 for “yes” and 0 for “no.”

#### Indications for cardiovascular disease

Medical records were queried for the presence and current treatment for the following five cardiovascular risks including hypertension (HTN), hypercholesterolemia, diabetes (DM), current tobacco use, and any indication for atherosclerotic cardiovascular disease (ASCVD; coronary heart disease, coronary artery stenosis, transient ischemic attack, ischemic stroke, and carotid artery stenosis, peripheral artery disease, aortic atherosclerotic disease). Each of these risks were coded 1 = present and 0 = absent.

### Statistical analyses

#### Self-reported memory concern

The relationship between self- reported memory concern and the presence of psychological distress (loneliness, anxiety, and depression) was assessed by calculating nominal contingency coefficients. The effect of self- reported memory concern on immediate and delay 3- word recall, DCTclock score, and DCR (combining word recall and DCTclock), and the four command/ copy DCTclock indices were assessed with 1-way analysis of variance (ANOVA) controlled for age, education, and sex.

#### Cardiovascular risk

The relationship between the DCR and DCTclock Score was assessed with a series of stepwise regression analyses. For each regression, the dependent variables were either the DCR or DCTclock Score; age, education, and sex were entered into block 1; and the five cardiovascular variables listed above were entered into block 2.

#### Factor analysis

Four factor analyses were conducted to extract the core variables that underlie digital clock drawing performance. The command and copy variables listed in Supplementary Table 1 for each index were analyzed. Factor analysis was used in the current research to reduce the large number of clock variables listed in Supplementary Table 1 in order to identify a smaller number of variables or features that explain digital clock drawing performance. Direct oblimin, non-orthogonal rotation (SPSS) was used because of presumed non- orthogonal, inter- dependence between clock variables within each clock index (Fabrigar et al., 1999).

## Results

### Demographics, DCR, and total clock drawing performance

As expected, NJISA participants were older; however, there were no differences for education or sex. NJISA participants obtained lower DCR and total clock drawing scores and presented with greater concern about memory and cardiovascular risks. Full statistics can be found in Appendix Table 1.

For the entire sample, the mean age and education were 72.43±12.46 and 14.52±2.61 years, respectively, and 66.40 percent of participants were female. A frequency distribution of DCR scores indicated that 59.60 percent of participants scored in the borderline or the impaired range (Table 2).

**TABLE 2:** Digital clock drawing and recall—frequency distribution.

DCR score	Frequency	Percent	Cumulative percent
0	16	9.4	9.4
1	15	8.8	18.2
2	37	21.8	40.0
3	38	22.4	**62.4**
4	30	17.6	80.0
5	34	20.0	100.0
Total	170	100.0	

Bold reflect the significant factor loadings for respective analyses.

### Self-reported memory concerns and psychological distress

Of the total sample, 162 participants were screened for self-reported memory concerns, and 68 (41.88%) participants screened positive for this problem. The entire sample (*n* = 199) was screened for psychological distress as described above. Concerning loneliness, anxiety, and depression, 42 (21.10%), 95 (47.73%), and 51 (25.62%) participants screened positive for these three problems. The association between self- reported memory concerns and loneliness (contingency coefficient = 0.209, *p* < 0.007), anxiety (contingency coefficient = 0.193, *p* < 0.012), and depression (contingency coefficient = 0.213, *p* < 0.020) were all significant. The three indicators of psychological distress were summed to create a single score (range 0–3). When self-reported memory concern was used as a grouping variable, participants reporting a concern about their memory also reported greater overall psychological distress [positive for memory concern, *M* = 1.25±1.14, negative for memory concern, *M* = 0.68±0.91; F(1, 160) = 12.34, η^2^ = 0.072, *p* < 0.001].

### Self-reported memory concerns, three-word recall, and the DCR

The effect of self- reported memory concerns on the total DCR score, 3- word immediate and delay recall, DCTclock score, and all DCTclock index scores was assessed with a series of 1- way ANOVAs controlling for age, education, and sex. No between-group differences were found for immediate or delay 3- word free recall. Participants with self- reported memory concerns obtained a lower DCR Score [positive for memory concern, *M* = 2.48±1.61, negative for memory concern, M = 3.26±1.54; F(1, 157) = 5.97, *p* < 0.016, η^2^ = 0.037], and a lower DCTclock score [positive for memory concerns, *M* = 50.16±29.16, negative for memory concerns, *M* = 61.66±26.73, F(1, 157) = 3.63, *p* < 0.050, η^2^ = 0.023].

**FIGURE 1:**
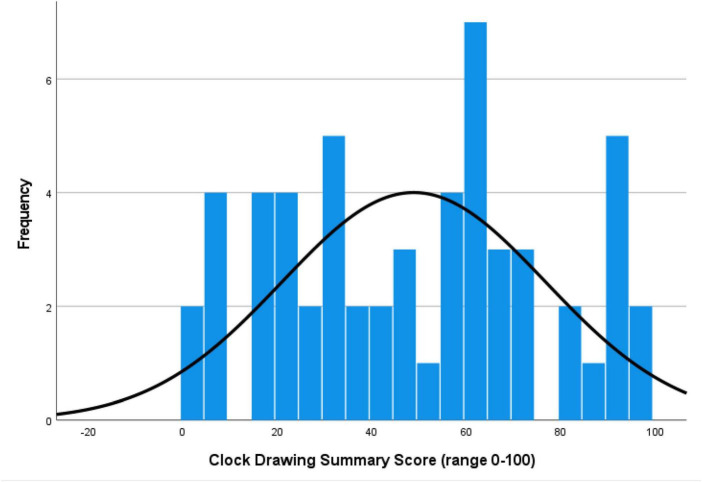
Frequency distribution of total command/ copy summary score: patients with self- reported memory concern.

Analyses examining for differences among the four command and four copy clock indices found that participants who were positive for self-reported memory concern obtained lower scores on the command Drawing Efficiency [positive for memory concern, *M* = −0.53+1.36, negative for memory concern, *M* = 0.02+1.37; F(1, 157) = 4.45, *p* < 0.036, η^2^ = 0.028], the command Simple and Complex Motor Operations [positive for memory concerns, M = −1.31±1.32; negative for memory concerns, M = −0.77±1.34; F(1, 157) = 4.73, *p* < 0.029, η^2^ = 0.034; Table 3]. However, as seen in Figure 1, the frequency distribution for self-reported memory concern and the total command/copy clock drawing score was bimodal, with many patients obtaining both low and high scores on this metric.

**TABLE 3:** Core clinical evaluation (means and standard deviations).

	Self- reported memory concern	No self- reported memory concern	Significance
Immediate 3- word free recall	2.84 (0.395)	2.69 (0.743)	0.224
Delay 3- word free recall	2.27 (1.01)	1.87 (1.16)	0.071
DCR	3.26 (1.54)	2.48 (1.61)	*p* < 0.016
Total command/ copy clock drawing score	61.66 (26.73)	50.16 (29.16)	*p* < 0.050

DCR, Digital Clock and Recall; ns, not significant.

### Cardiovascular risk, DCR, and total command/copy clock drawing

The stepwise regression examining the association between the DCR, and cardiovascular risks found that demographic variables entered in block 1 were significant (*R* = 0.415, *R*^2^ = 0.172, df = 3/188, *F* = 13.05, *p* < 0.001). In block 2 only ASCVD entered the final model (*R* = 0.438, *R*^2^ = 0.192, df = 1/187, *F* = 4.46, β = −0.143, *p* < 0.036) such that a *better* DCR score was associated with the *absence* of ASCVD.

The stepwise regression for the DCTclock Score resulted in a similar finding. Demographic variables were significant in block 1 (*R* = 0.422, *R*^2^ = 0.178, df = 3/188, *F* = 13.54, *p* < 0.001). In block 2, only treatment for DM entered the final model (*R* = 0.444, *R*^2^ = 0.197, df = 1/187, *F* = 4.49, beta = −0.140, *p* < 0.035) such that *better* clock drawing performance was associated with the *absence* of DM.

### Clock drawing factor analysis

Factor analysis for the *Drawing Efficiency* command/copy variables produced a 4- factor solution (80.08 percent of variance explained, communalities 0.694–0.801). Factor 1 was comprised of both command/ copy ink length and size (33.41 percent variance), factor 2 was comprised of command total pen strokes and total extraneous pen strokes (24.93 percent of variance), factor 3 (11.50 percent of variance) was comprised of command/ copy total clock drawing time to completion, and factor 4 consisted of copy total pen strokes and total extraneous pen strokes (10.22 percent of variance).

The analysis of *Simple and Complex Motor* variables produced a 2- factor solution (83.14 percent of variance, communalities 0.767–0.896). Factor 1 was comprised of all variables measuring how quickly pen strokes were drawn (66.63 percent of variance). Factor 2 was comprised of variables assessing command and copy oscillatory motion (16.51 percent of variance).

The analysis of *Information Processing Speed* variables also produced a 2- factor solution accounting for 89.66 percent of variance (communalities 0.774–0.955). Factor 1 contained all command variables (65.66 percent of variance), while factor 2 contained all copy variables (23.99 percent of variance).

The analysis of the *Spatial Reasoning* variables produced a 3- factor solution (58.97 percent of variance, communalities 0.414–0.718). Factor 1 consisted of command/ copy clock face circularity, and the accuracy of placement of numbers and the clock hands within the clock face (28.27 percent of variance). Factor 2 was related to the horizontal position of both command/ copy drawings on the iPad (17.37 percent of variance), and factor 3 was related to the vertical position of both command/ copy drawings on the iPad (13.32 percent of variance; Tables 4–7).

**TABLE 4:** Digital Clock Drawing—Drawing Efficiency: factor analysis.

	Factor 1	Factor 2	Factor 3	Factor 4
Drawing Efficiency Command stroke count	−0.043	0.857	0.059	0.267
Drawing Efficiency Copy stroke count	0.009	0.187	0.149	**0.884**
Drawing Efficiency Command total time to completion	0.018	0.377	−**0.744**	−0.159
Drawing Efficiency Copy total time to completion	0.000	−0.110	−**0.915**	0.126
Drawing Efficiency Command ink length	**0.885**	−0.190	0.031	0.151
Drawing Efficiency Copy ink length	**0.849**	0.163	0.160	−0.168
Drawing Efficiency Command size	**0.865**	−0.087	−0.128	0.133
Drawing Efficiency Copy size	**0.863**	0.126	−0.068	−0.091
Drawing Efficiency Command noise	0.062	**0.833**	−0.135	0.005
Drawing Efficiency Copy noise	0.041	0.042	−0.345	**0.680**

Bold reflect the significant factor loadings for respective analyses.

**TABLE 5:** Digital clock drawing—Simple and Complex Motor Operations: factor analysis.

	Factor 1	Factor 2
Motor Command average speed	0.915	0.113
Motor Copy average speed	**0.956**	−0.014
Motor Command maximum speed	**0.914**	−0.014
Motor Copy maximum speed	**0.958**	−0.195
Motor Command initiation speed	**0.863**	0.069
Motor Copy initiation speed	**0.892**	0.007
Motor Command termination speed	**0.857**	0.114
Motor Copy termination speed	**0.899**	−0.013
Motor Command oscillatory motion	0.041	**0.922**
Motor Copy oscillatory motion	−0.013	**0.925**

Bold reflect the significant factor loadings for respective analyses.

**TABLE 6:** Digital clock drawing—Information Processing Speed: factor analysis.

	Factor 1	Factor 2
Information Processing Speed Command average latency	0.904	0.079
Information Processing Speed Copy average latency	0.083	**0.894**
Information Processing Speed Command average latency variability	**0.982**	−0.010
Information Processing Speed Copy average latency variability	−0.045	**0.992**
Information Processing Speed Command long latency	**0.974**	0.008
Information Processing Speed Copy long latency	−0.008	**0.970**
Information Processing Speed Command latency count	**0.878**	0.002
Information Processing Speed Copy latency count	0.045	**0.862**
Information Processing Speed Command longest latency	**0.988**	−0.055
Information Processing Speed Copy longest latency	−0.049	**0.979**

Bold reflect the significant factor loadings for respective analyses.

**TABLE 7:** Digital clock drawing—Spatial Reasoning: factor analysis.

	Factor 1	Factor 2	Factor 3
Spatial Reasoning Command face circularity	0.625	0.125	−0.080
Spatial Reasoning Copy face circularity	**0.718**	−0.248	−0.131
Spatial Reasoning Command component placement	**0.759**	0.079	−0.199
Spatial Reasoning Copy component placement	**0.743**	−0.132	−0.317
Spatial Reasoning Command vertical placement	0.088	0.121	−**0.855**
Spatial Reasoning Copy vertical placement	0.312	0.014	−**0.792**
Spatial Reasoning Command horizontal placement	0.062	**0.772**	−0.211
Spatial Reasoning Copy horizontal placement	−0.084	**0.768**	0.052

Bold reflect the significant factor loadings for respective analyses.

A series of ANOVAs were conducted to assess how the 11 factors derived from the four analyses described above were related to cardiovascular risk, psychological distress, and self- reported memory concerns. Greater cardiovascular risk resulted in command and copy drawings that were larger [Drawing Efficiency- factor 1; F(1, 186) = 6.80, *p* < 0.010, η2 = 0.035]; and drawn with greater command and copy oscillatory motion [Simple/Complex Motor- factor 2; F(1, 186) = 5.22, *p* < 0.027, η^2^ = 0.027]. No additional differences were found.

## Discussion

In the current research a large group of ambulatory care patients completed the CCE, a novel, digital cognitive assessment. As described above, all participants were assessed as part of their routine medical care and were living independently in the community. None of these patients had been clinically diagnosed with MCI or dementia. Interestingly, many of these patients scored in the range for borderline or cognitive impairment on the CCE, emphasizing the value of population screening, and the likely high number of under-recognized instances of age-related cognitive impairment.

The current research documented statistical relationships between performance on the CCE, patients’ memory concerns, psychological distress, and cardiovascular risk factors (Vintimilla et al., 2022). Of those screened for self-reported memory impairment, 41 percent reported concerns about declining memory; and 30 percent of patients screened positive for psychological distress involving loneliness, anxiety, or depression. These aspects of psychological distress are common in older adults (Vieira et al., 2014; Gundersen and Bensadon, 2023). Moreover, these problems are often under-diagnosed and under-treated, associated with emotional suffering (Hybels and Blazer, 2003), and increasing healthcare expenditure (Katz, 1996; Unützer et al., 2009).

Both self-reported concern about memory impairment and psychological distress have been shown to be related to the eventual emergence of dementia. For example, in a comprehensive review of prior research, Jonker et al. (2000) found that in community-based individuals, self-reported memory concerns predicted the onset of dementia within 2 years. Jiang et al. (2022) found that psychological distress was an early warning sign and predictor of dementia such as AD. Zullo et al. (2021) studied a group of community-dwelling, dementia-free individuals, and found that 18.5% of their sample reported concerns about memory. Zullo et al. (2021) also noted that self- reported memory concern was associated with depression, as well as lower performance on tests that assessed episodic memory and verbal fluency, a test like the clock drawing test that also measures executive abilities.

In the current research, patients presenting with self-reported memory concerns scored lower on the DCTclock total command/ copy summary score and the DCTclock command Drawing Efficiency and Simple/Complex Motor indices. Reduced performance on these indices suggests some compromise in gross constructional abilities and slowness in the production of pen strokes. However, the frequency distribution of the total command/ copy summary score for patients reporting concerns about memory revealed at least a bimodal distribution. This means that patients reporting concerns about their memory obtained both impaired and intact scores on this measure.

Concern about memory, along with an *intact* score on this metric could identify the individual as among the “worried well.” For these patients, psychological problems or other factors could be present that may not necessarily be related to an emergent ADRD illness. In these situations, referral for behavioral health treatment could be indicated. On the other hand, patients expressing both concern about their memory along with an impaired score on this metric could suggest the emergence of a more serious problem. For these patients, further diagnostic work-up for an underlying medical problem and/or an ADRD illness might be considered. These data, as available from the CCE, provide the clinician with the necessary information for targeted and meaningful clinical decision making.

Recently, long-standing, albeit treated cardiovascular disease, has emerged as a risk for the eventual emergence of ADRD (see Emrani et al., 2020; Vintimilla et al., 2022 for a review). Moreover, the presence of cardiovascular risks is associated with declining neuropsychological test scores. For example, De Anda-Duran et al. (2022) examined a sample of research participants from the Bogalusa Heart Study (Berenson, 2001). Having a carotid intima-media thickness (c-IMT) > 50th percentile was inversely associated with a demographically standardized global cognitive score. Also, greater c-IMT was associated with poorer performance on tests that assessed executive and verbal episodic memory abilities. Lamar et al. (2021) studied a sample of community volunteers. Their neuropsychological test scores were subjected to a latent profile analysis. Four groups emerged, including a group with lower scores on executive tests. Similar to the results in the current research, participants with dysexecutive difficulty also presented with higher fasting glucose and hemoglobin A1c than other groups. The association between the CCE test parameters and treatment for DM and the presence of ASCVD, as described above, increases the clinical effectiveness and utility of the CCE to screen for vascular co-morbidities that might both aggravate and potentiate ADRD. Our results suggest that the CCE may be used to monitor the potential impacts of cardiovascular disease on cognition. All of this information can inform clinical decision-making resulting in improved patient care.

The association between self- reported memory problems and psychological distress, cardiovascular risks, and lower performance on neurocognitive tests, such as clock drawing and three-word recall, is likely highly nuanced and complex. The data reported above should not necessarily be interpreted to suggest simple, linear relations between these problems. Moreover, while instances of self- reported memory difficulty, along with elevated cardiovascular risks, psychological distress, and subsequent lower CCE neurocognitive scores are clinically useful, it remains to be determined how this constellation of clinical symptoms is related to either Alzheimer’s disease pathology, or the eventual emergence of either MCI or a dementia. Paradise et al. (2011) studied a large sample of middle- aged people with no history of affective illness or stroke. In this sample, psychological distress was strongly, but cardiovascular risks were only weakly associated with self- perceived memory problems. Still, these researchers suggested that psychological distress could be mediating the association between cardiovascular risks and self- perceived memory problems. Nonetheless, in an older population it could be that cardiovascular risks may take on a more prominent role regarding lower performance on neurocognitive tests.

Underlying the four DCTclock indices are 44 variables assessing a host of graphomotor and time-based behavior. Factor analyses were conducted to better understand and extract a core group of variables that underlie digital clock drawing behavior. The factor analysis of the command/ copy Drawing Efficiency variables yielded a four-factor solution. The results in Table 4 suggest that Drawing Efficiency might best be understood as assessing *general or gross constructional abilities* necessary for successful clock drawing. For example, factor 1 appears to assess the size of both command and copy clock drawings. The variables loading in factors 2 and 4 assessed the number of productive and extraneous pen stokes produced, respectively. The observation that productive and extraneous pen strokes load on separate factors is consistent with prior clock drawing research (Libon et al., 1993, 1996; Cosentino et al., 2004) suggesting divergent, but complementary behavior underlies clock drawing to command versus clock drawing to copy. Factor 3 is related to gross, overall time to completion for both the command and copy clock drawings.

The factor analysis of the Simple and Complex Motor index variables produced a two-factor solution. Table 5 shows that most of these variables measure *graphomotor or drawing speed* with which individual pen strokes are drawn. However, the command and copy oscillatory motion variables loaded on its own factor. Regarding clinical decision making, the emergence of oscillatory behavior could suggest a wide number of problems. For example, arthritis could be present. However, other problems including unwanted medication side effects, as well as an emergent idiopathic movement disorder should be considered.

The analysis of variables displayed in Table 6 from the Information Processing Speed index also produced a two-factor solution. All command variables loaded on one factor while all copy variables loaded on the second factor. These variables measure the latency or pauses in between the production of pen strokes. This behavior can be described as “*think-time*” as compared to the production of actual pen strokes or “*ink-time*.” In an analysis of participants from the Framingham Heart Study, Piers et al. (2017) found age-related differences for selected “*think*” versus “*ink*” parameters. Also, for some of these variables, greater between-group differences were found in the copy versus the command test condition. As described above, Dion et al. (2020) found that lengthier pauses between the production of pen strokes, *termed intra- component latency*, was negatively associated with worse performance on neuropsychological tests that assessed working memory and Information Processing Speed. In another analysis of participants from the Framingham Heart Study, Yuan et al. (2022) found that similar behavior was associated with greater MRI white matter alterations. Thus, a low score on this index could signal the need for additional investigation into the emergence of problems related to an ADRD illness.

Interestingly, *think- time* command and copy variables loaded on separate factors, again, suggesting that divergent abilities underlie clock drawing to command versus clock drawing to copy. This supposition is consistent with clinical experience. For example, in the copy test condition there can be a great deal of visual scanning back and forth from the patients drawing to the provided model. This behavior is obviously absent in the command test condition.

The factor analysis from the Spatial Reasoning Index yielded a three-factor solution and appears to be related to visual reasoning and mental planning. The results displayed in Table 7 found that the circularity of the command/copy clock face, and the accuracy of how well the numbers and clock hands were drawn within the clock face all loaded on a single factor. The horizontal and vertical placement of the drawing in the space provided loaded on other factors. Using a relevance factor variational autoencoder (RF-VAE), a deep neural network, Bandyopadhyay et al. (2022) found that an irregularly drawn clock face was able to distinguish between dementia versus non- dementia patients. As described above, Dion et al. (2022) found that errors in number placement was associated with poorer performance on selected neuropsychological tests and negatively associated with connectivity from the basal nucleus of Meynert (BNM) to the anterior cingulate cortex (ACC). Thus, the results of this series of factor analyses suggest that approximately ten variables or features underlie digital command and copy clock drawing behavior. Finally, when groups were constructed to express greater versus less cardiovascular risk, significant motor problems were found. Specifically, participants with greater cardiovascular risk produced command and copy drawings that were larger with greater oscillatory motion.

The current research is not without limitations. The results reported above are constrained because of the lack of greater ethnic/racial diversity. Also, persons with minimal formal education need to be assessed. Tobacco and alcohol use are also related to cardiovascular risk but were not tallied because of potentially unreliable participants’ self-report. Thus, cardiovascular risk could be underrepresented in this sample. Finally, the assessment of psychological distress was very brief. As such, both the presence and degree of psychological distress could also be underrepresented. Nonetheless, the current research has many strengths. The CCE requires no more time to administer than standard paper and pencil cognitive screening tests such as the MMSE or MoCA. Also, because the test is administered and scored automatically using an iPad and accompanying software, scoring subjectivity, inherent in traditional paper and pencil tests, is not present and reliability is greatly increased.

Unlike other brief mental status tests, the CCE can also assess how self-reported memory concerns, psychological distress, and neurocognitive abilities are related to each other and to cardiovascular risk. The amalgamation of all these factors provides healthcare providers with actionable information about their patients for targeted and meaningful healthcare decision making.

## Data availability statement

The original contributions presented in this study are included in this article/Supplementary material, further inquiries can be directed to the corresponding author.

## Ethics statement

The studies involving humans were approved by the Rowan University, Institutional Review Board. The studies were conducted in accordance with the local legislation and institutional requirements. The participants provided their written informed consent to participate in this study.

## Author contributions

DL: Conceptualization, Data curation, Formal analysis, Methodology, Writing – original draft, Writing – review and editing. EM: Conceptualization, Formal analysis, Methodology, Writing – review and editing. SC: Conceptualization, Methodology, Writing – review and editing. CP: Methodology, Writing – review and editing, Conceptualization, Validation. RS: Conceptualization, Methodology, Writing – original draft, Writing – review and editing. MV: Conceptualization, Data curation, Methodology, Writing – review and editing, Validation. TG: Conceptualization, Data curation, Methodology, Writing – review and editing. AO-U: Conceptualization, Data curation, Writing – review and editing. LP: Data curation, Writing – review and editing. RN: Writing – review and editing. ST: Formal analysis, Methodology, Writing – review and editing. JG-O: Writing – review and editing. AP-L: Writing – review and editing.

## Appendix

**APPENDIX TABLE 1:** Demographic and clinical evaluation (means and standard deviations).

	Family medicine	NJISA	Significance
Age	61.02 (14.57)	77.01 (7.75)	*t* (197) = 10.03; *p*< 0.001
Education	14.32 (2.25)	14.60 (2.75)	*t* (197) = 0.688; *p*< 0.492
Percent female	73.7%	64.8%	*X*^2^ (1) = 1.46; *p*< 0.246
Self- reported memory concerns	25.0%	47.2%	*X*^2^ (1) = 6.09; *p*< 0.016
DCR (range 0–5)	3.75 (1.31)	2.71 (1.55)	*t* (197) = 4.46; *p*< 0.001
Total command/ Copy clock drawing score (range 0–100	72.33 (23.09)	51.74 (27.16)	*t* (197) = 5.03; *p*< 0.001
Cardiovascular risk	1.69 (1.38)	2.10 (1.24)	*t* (197) = 2.03; *p*< 0.044

DCR, digital clock and recall; ns, not significant.
