# Supplementary material for: Using digital assessment technology to detect neuropsychological problems in primary care settings
Source: Front Psychol. 2023 Nov 17;14:1280593. doi: 10.3389/fpsyg.2023.1280593 (PMC10693332; doi:10.3389/fpsyg.2023.1280593)
Supplement: Supplementary file 1 [file Table_1.pdf]

Supplemental Table 1 – Digital Drawing Indices and Variables

Drawing Efficiency

1. Stroke Count Conformity - The deviation from the expected number of pen strokes in the drawing.

2. Total Time To Completion - The total time spent completing the drawing measured from the first touch of the pen on the paper to the last pen lift off the paper.

3. Total Ink Length - The sum, in millimeters, of all pen stroke lengths used in the drawing.

4. Drawing Size - The size, in millimeters, of the clock face circle.

5. Drawing Process Efficiency - A relative measure that combines Ink Length and Total Time.

6. Noise - A measure of the drawing that includes non-standard pen strokes, cross-outs, and overwriting.

Simple and Complex Motor Key Cognitive Features

1. Percent Ink Time - The percentage of the test time spent actively drawing with the pen on the paper.

2. Average Speed - The average speed of the pen for all pen strokes used during the drawing of the clock face.

3. Max Speed - The maximum speed of the pen on the page during the drawing of the clock face.

4. Initiation Speed - The speed of the pen when beginning to draw the clock face.

5. Termination Speed - The speed of the pen when finishing the clock face.

6. Oscillatory Motion - A measure of how much the motion of the pen deviates from a smooth pen motion during the drawing process.

Information Processing Key Cognitive Features

1. Percent Think Time - The percentage of the test time spent "thinking" (i.e., holding the pen off the page but not actively drawing) measured from the first touch of the pen on the paper to the last pen lift off the paper.

2. Average Latency **-** The average duration of the latencies between each pen stroke.

3. Latency Variability **-** The variability in the latencies throughout the drawing process.

4. Relative Long Latency **–** A measure of the differences among the average latency and the longer latencies within this drawing.

5. Long Latency Count **-** The total number of latencies in the drawing that are notably longer than the normative sample standard.

6. Longest Latency **-** The duration of the longest latency in the drawing.

Spatial Reasoning Key Cognitive Features

1. Clock Face Circularity - A measure of the roundness of the clock face circle.

2. Component Placement – A measure of the spatial relationships among the drawing components.

3. Vertical Spatial Placement – A measure of the vertical position of the drawing on the page.

4. Horizontal Spatial Placement – A measure of the horizontal position of the drawing on the page.
